# Morphological Seed Traits Predict Early Performance of Native Species to Pelletized Seed Enhancement Technologies

**DOI:** 10.3390/plants13162256

**Published:** 2024-08-14

**Authors:** Samantha E. Andres, Paige E. Lieurance, Charlotte H. Mills, Sasha G. Tetu, Rachael V. Gallagher

**Affiliations:** 1 Hawkesbury Institute for the Environment, Western Sydney University, Penrith, NSW 2751, Australia; 2 AirSeed Technologies, Sydney, NSW 2000, Australia; 3 Centre for Ecosystem Science, School of Biological, Earth and Environmental Sciences, UNSW Sydney, Kensington, NSW 2052, Australia; 4 School of Natural Sciences, Macquarie University, Sydney, NSW 2109, Australia

**Keywords:** seed-based restoration, plant conservation, nature repair, germination biology, trait ecology, seed science, seed handling

## Abstract

Native seeds are a finite resource, and their inclusion in revegetation is vital for supporting restoration outcomes that are both effective and scalable. Pelletized seed enhancement technologies (SETs) offer a promising solution to improve seed use efficiency in ecological restoration. Yet, knowledge of how diverse suites of native species perform when pelletized is required to optimize the application of SETs to the many species and ecosystems where restoration is required. Using a greenhouse trial of 64 Australian plant species, we assessed species performance to pelleting by evaluating (1) overall species amenability to pelleting based on total emergence and survival and (2) how pelleting modifies the rate of emergence based on average time to emergence, emergence rate index, and time spread of emergence. We investigated the potential for using morphological seed traits (seed endosperm:seed ratio, seed length, seed area, and seed coat thickness) to predict performance outcomes, by identifying traits that may aid in the prediction of species amenability to pelleting and emergence speed when pelletized. We found that some species demonstrate high amenability to pelleting and that pelleting can modify the emergence rates for many species. This work advances our understanding of the applicability of SETs for diverse native species, demonstrating the application of such technologies for meeting ecological restoration goals.

## 1. Introduction

Revegetation using native seeds is often used to reinstate native plant communities as part of ecological restoration. However, low seedling emergence is often reported [1,2], with average plant establishment estimated to be as low as 5–10% of the total seed sown [3,4,5]. As a result, high densities of native seed—between 600 and 20,000 seeds per m^2^ (depending on the focal species and locale) are required when conducting seed-based revegetation [6,7,8]. Seed provision at this scale is expensive and places pressure on the native plant industry and wild populations, putting them at risk of overharvesting [4,9,10]. Likewise, if diverse native seeds in the quantities required to undertake restoration cannot be procured, global restoration targets such as the UN Decade on Restoration, Kunming-Montreal Global Biodiversity Framework and Bonn Challenge may not be effectively met [11,12,13]. Therefore, it is essential to optimize the use of native seed in ecological restoration to support both scalable and cost-effective revegetation outcomes, whilst ensuring the sustainable use of germplasm [3,4,14].

Seed enhancement technologies (SETs) are used to improve the delivery of seed to a site while improving germination and seedling establishment, making better use of the finite stocks of the seed available for restoration. SETs, which include the artificial pre-treatment or coating of seeds prior to planting, were initially developed to improve early emergence, growth, and establishment and to reduce seed wastage in agricultural species [15,16]. SETs are now being used in restoration to control seed handling, increase the density and growth of seedlings, and reduce the losses attributed to predation or displacement from a site, thus maximizing seed use efficiency [17,18,19,20]. SETs can also be combined with novel precision planting methods such as of unmanned aerial vehicles (also known as drones), offering a scalable, cost-effective solution to restoration [14,21]. 

To date, the application of SETs across a range of native plant species has yielded mixed or species-specific results, suggesting further research is required to optimize the application of this technology [22,23]. In a recent study exploring the application of pelletized seeds (a type of SET where seeds are attached to or encapsulated in an organic substrate) to native species, seed mass was found to predict species response to pelleting and influence emergence outcomes [23]. Given the promise of trait-based approaches toward improving the application of SETs to native species, it is valuable to assess the inclusion of other seed traits for predicting species performance using SETs. 

Seed-based restoration faces multiple demographic barriers (e.g., germination, emergence, establishment) that must be overcome for successful establishment [24,25]. Such barriers to emergence pose a particularly strong filter on plant recovery outcomes in restoration and frequently limit the success of many revegetation efforts using native seed [26,27,28]. As native species can vary substantially in the set of conditions required for emergence [29], there is a need to understand how SETs affect regenerative characteristics across diverse native species to assess the suitability and cost-effectiveness of their use. The assessment of performance characteristics such as total emergence, emergence speed, and survival for different native species can serve as early indicators of the general ‘amenability’ of a species to SET compared to bare seeds [25,30].

Morphological seed traits have been previously linked to emergence characteristics in restoration settings [31,32,33]. For example, seed mass (as a surrogate for seed size) is frequently associated with emergence, where larger seeds tend to show greater total emergence and survival compared to smaller seeds, likely owing to larger nutrient reserves (endosperm to seed ratio (E:Sarea)) [34,35,36,37]. Species with thicker seed coats tend to form more persistent seedbanks in the soil and exhibit patterns of delayed emergence or dormancy relative to species with thinner seed coats due to the increased time it takes for imbibition or for suitable conditions to alleviate dormancy and cue germination [38,39,40]. Studies have also observed relationships between seed length and shape variance, where longer, nonspherical seeds tend to emerge more rapidly than shorter, rounder seeds [31,41]. Elongated and narrow seeds may also exhibit rapid emergence as a response to their lower likelihood of burial and long-term persistence in the soil [41,42,43]. 

**Figure 1:**
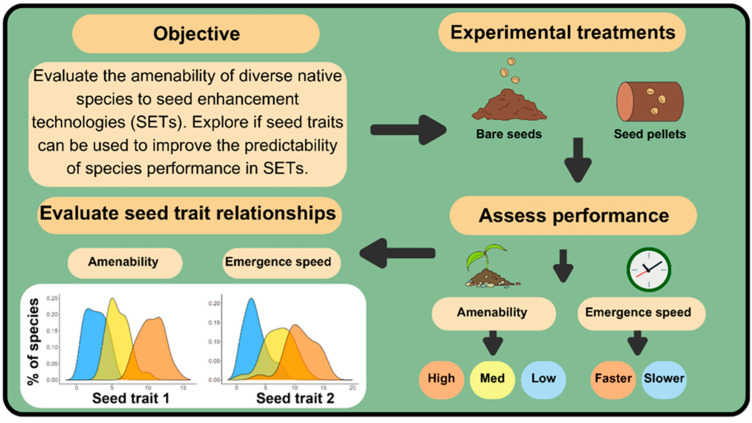
Conceptual diagram outlining the experimental study objectives and design.

Here, we compared the performance of a diverse suite of 64 native Australian plant species grown from bare seed to that of seed prepared using a pelletized form of SET (referred to as ‘pellets’ hereafter) (Figure 1) to evaluate the application of this common type of SET for use in revegetation. Species performance to pelleting was evaluated using two metrics of general amenability: (1) total seedling emergence and (2) survival; and three metrics relating to the speed of emergence: (1) time to emergence, (2) emergence rate index, and (3) time spread of emergence. Seed morphological traits (seed endosperm:seed ratio, seed length, seed area, and seed coat thickness) were quantified for all species to explore the degree to which performance of pellets (emergence speed and overall amenability to pellets) can be predicted by seed traits. Our aims for this study were to

1. Characterize the amenability of 64 native species subject to pelleting by identifying species with comparable (or higher) total emergence and survival when pelletized relative to bare seeds;
2. Quantify the degree to which pelletization modifies emergence speed across species;
3. Quantify whether morphological seed traits can be used to predict species performance (amenability and emergence speed) to pelleting across a diverse range of species.

## 2. Results

At the end of the experiment (19 weeks), seedlings emerged from 1616 (47%) pellets and 1669 (49%) bare seeds. Total emergence (%) ranged from 0 to 100%, with an average of 52% total emergence from bare seeds and 50% total emergence from pellets. We observed 0% total emergence for six species (irrespective of treatment) (*Caesia parviflora*, *Eucalyptus oblonga*, *Eragrostis brownii*, *Styphelia sieberi*, *Solenogyne bellioides*, *Vittadinia cuneata*) (Table 1). Survival ranged from 0 to 100%, with an average survival of 83% for bare-seeded individuals and 79% for individuals grown from pellets (Table 1). Of the replicates that successfully emerged, the average time to emergence ranged from 7 to 127 days (1 to 18 weeks), with the rate of emergence (emergence rate index) ranging from 0.02 to 2.07 individuals per day and the time spread of emergence ranging from 0 to 120 days (Table 1).

### 2.1. Emergence and Survival 

**Figure 2:**
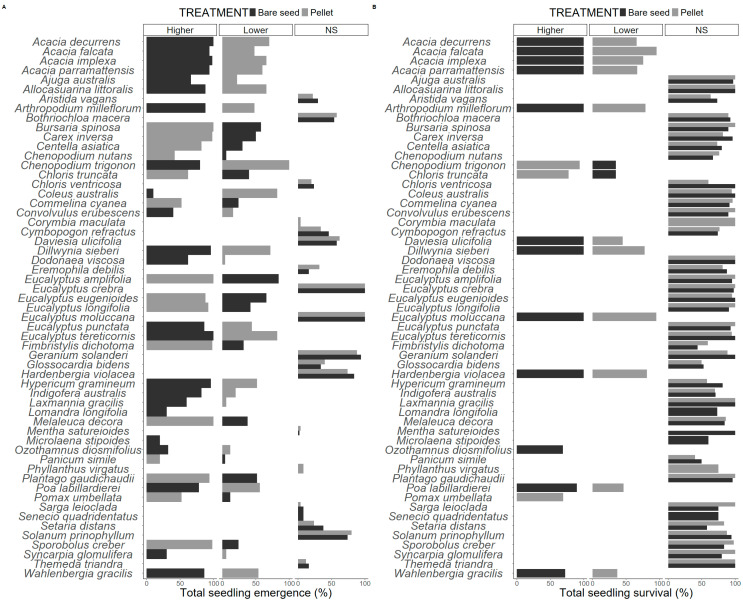
(**A**) Total seedling emergence (%) and (**B**) survival (% of emerged seedlings that survived) for bare and pelleted seed treatments (n = 50 replicates per treatment, per species). Within species, data for treatments significant at *p* < 0.05 (Pearson’s chi squared test of independence) are represented in the following panels (higher, lower), whereas data of species with nonsignificant differences are represented in the ‘NS’ panel.

Of the 64 species included in this study, 29% (17 species) responded with significantly higher total emergence from pellets relative to bare seeds (Figure 2; Table S3). Conversely, 36% (21 species) responded with significantly lower total emergence from pellets relative to bare seeds (Figure 2; Table S3). The remaining 34% (20 species) of species tested in this study exhibited comparable emergence across both treatments (Figure 2; Table S3). We observed no significant difference in survival among individuals grown from either treatment for 70% of the tested species (37 species) (Figure 2; Table S2). Survival from pellets was significantly higher for 7% (4 species) and significantly lower for 23% (12 species) (Figure 2; Table S3).

### 2.2. Emergence Speed

Time to emergence significantly differed between bare and pelleted seed treatments (*p* < 0.001); however, there was considerable variation within species. Within species, time to emergence for bare seeds relative to pellets was significantly shorter for 17 of the 53 species analyzed (Table S4). Conversely, we observed significantly shorter time to emergence from pellets relative to bare seeds in 11 of the 53 species analyzed (Table S4). The emergence rate index (*p* = 0.074) and time spread of emergence (*p* = 0.902) did not differ between treatments. However, differences within species were observed for these metrics (Table 1). Of all the species analyzed, 45% (24 species) exhibited a higher emergence rate index when grown from pellets relative to bare seeds, 51% (27 species) exhibited a lower emergence rate index from pellets relative to bare seeds, and 4% (2 species) exhibited no difference between treatments. Additionally, 40% (21 species) exhibited a faster time spread of emergence, 53% (28 species) exhibited a slower time spread of emergence, and 7% (4 species) exhibited no difference as pellets or bare seeds.

### 2.3. Ranking Species Performance in Pellets 

Of the 53 species where emergence and survival were analyzed, 57% (30 species) exhibited high amenability to the pellet recipe tested in this study (Table S2). These species exhibited similar or significantly higher emergence and early survival (Figure 2; Table 1). Medium (similar or improved outcomes for one of the two response variables) and low (similar or improved outcomes for neither response variables) amenability to the pellet recipe were observed for 28% (15 species) and 15% (8 species) of species, respectively (Table S2). Additionally, 34% (18 species) of the species responded with faster emergence in pellets relative to bare seeds (faster emergence for at least two of the three response variables) (Table S2). Meanwhile, 43% (23 species) of the species tested responded to pellets, with slower emergence in pellets relative to bare seeds (slower emergence for at least two of the three response variables) (Table S2). The remaining 23% (12 species) exhibited no difference in emergence speed when sown as bare seeds or in pellets (Table S2). 

### 2.4. Morphological Seed Traits and Performance in Pellets

**Figure 3:**
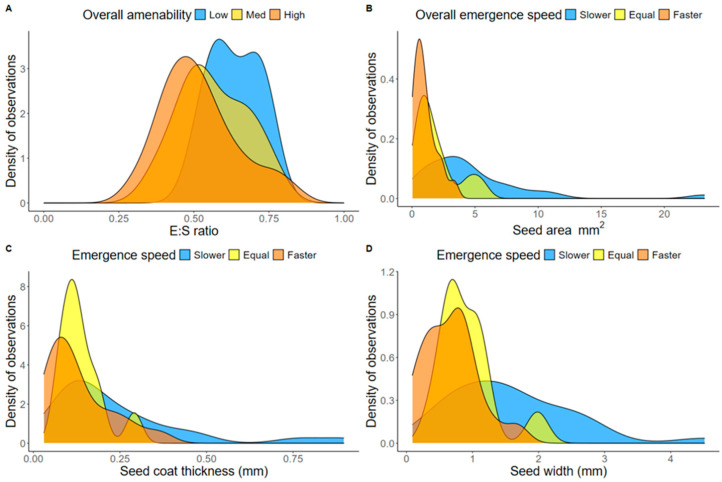
Species performance with the pellet recipe tested in this study for 53 native species, summarized by morphological seed trait: (**A**) the distribution of seed E:Sarea based on overall amenability ranking, (**B**) the distribution of seed area based on emergence speed ranking, (**C**) the distribution of seed width based on emergence speed ranking, and (**D**) the distribution of seed coat thickness based on emergence speed ranking.

The morphological seed traits explained the trends associated with the overall species performance in pellets (Table S2; Figure 3A) as well as the emergence speed from pellets relative to bare seeds (Table S2; Figure 3B–D). Specifically, seed E:Sarea differed across species with different amenability rankings (*p* = 0.034), such that highly amenable species had a seed E:Sarea 0.11 times lower than that of species with low performance (*p* = 0.030) (Figure 3A). Additionally, seed area predicted overall emergence speed (*p* = 0.001), where seeds from species that were slower to emerge in pellets were 3.73 mm^2^ larger than faster-to-emerge species (*p* = 0.001) (Figure 3B). Seed width also predicted emergence speed (*p* < 0.001), where species with wider seeds (0.91 mm wider) demonstrated slower emergence in pellets compared to species with faster emergence (*p* = 0.003) (Figure 3C). Additionally, species with slower pellet emergence had seeds that were 0.67 mm wider than those of species where equal emergence speed was observed between treatments (*p* = 0.030). Finally, seed coat thickness predicted overall emergence speed (*p* = 0.036), where species with thicker seed coats (0.11 mm thicker) exhibited marginally slower emergence from pellets compared to species with faster emergence (*p* = 0.049) (Figure 3D).

**Figure 4:**
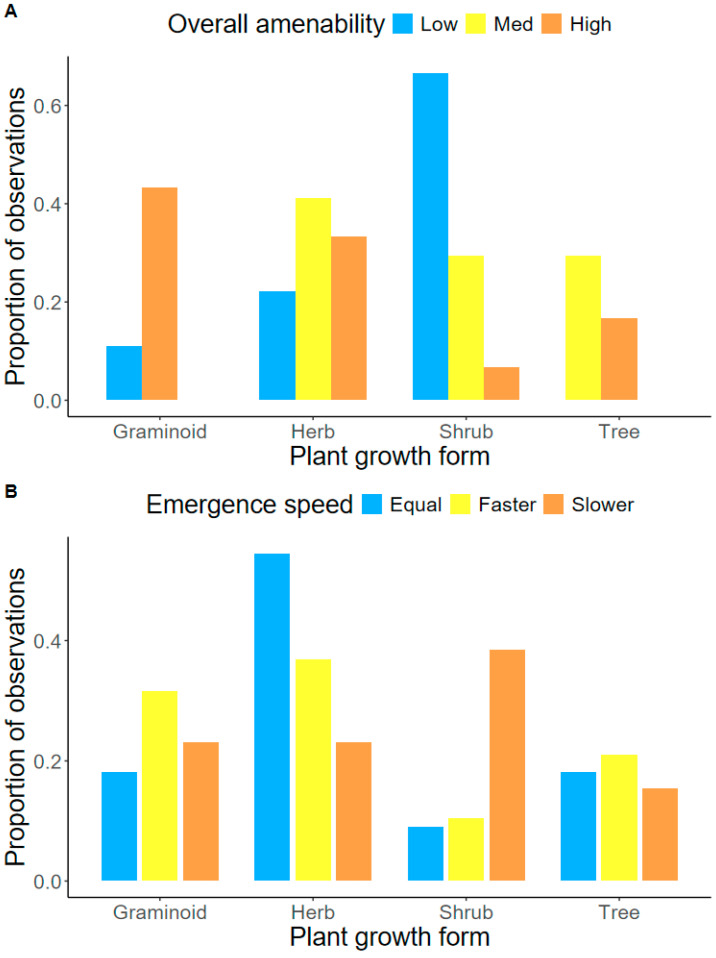
Species amenability to the pellet recipe tested in this study summarized by plant growth form (tree, shrub, herb, graminoid). (**A**) Overall amenability (low, medium, high) with pellets, and (**B**) emergence speed from pellets (faster, equal, slower) relative to bare seeds among the 53 species analyzed in this study.

Among the studied species, graminoids exhibited the best performance (i.e., the highest proportion of “high-amenability species”) of all growth forms with the tested pellet recipe, and shrubs exhibited the least (i.e., the highest proportion of “low amenability” species) (Figure 4A). The tree species included in this study generally exhibited medium or high performance in pellets; meanwhile, herbaceous plant growth forms demonstrated mixed performance (Figure 4A). The overall emergence speed from pellets exhibited fewer trends across plant growth forms relative to bare seeds, with the exception of shrubs, which generally exhibited slower emergence when grown using the pellets recipe tested in this study relative to bare seeds (Figure 4B).

## 3. Discussion

Across most species, we observed variation in the speed of emergence among seedlings grown from pellets relative to bare seeds, consistent with the findings of past studies [30,44]. Emergence was delayed for species grown from pellets in 43% of the species trialed. However, we also observed faster emergence from pellets for 34% of the species. Understanding how SETs, such as pellets, modify species’ regenerative attributes (such as emergence) is important for restoration practitioners aiming to optimize the delivery of seed to a site to improve plant establishment [34]. For example, species with high total emergence or postemergence survival might be preferable to use when conducting primary seeding efforts to encourage seedling establishment [24,45,46]. Additionally, species that emerge quickly after sowing may be advantageous to include in early restoration, as evidence frequently suggests that giving ‘priority’ to early emergers can occupy the open niche space available for colonization of nontarget weedy species [27,47,48,49]. Similarly, planting seeds with a larger spread of emergence over time might be desirable for sites located in regions with unpredictable weather to maximize the likelihood of emergence occurring during a window of suitable environmental conditions [29,50].

We demonstrate that a range of morphological seed traits can be used to improve the predictability of how species might respond to pelletized SETs. Seed E:Sarea was the strongest predictor of species amenability to pelletization, where species with a low E:Sarea demonstrated high amenability to pelleting. This may be attributed to the microenvironment provided by the water-retaining nature of the pellet. Indeed, past studies demonstrated that species with a low E:Sarea are frequently found in habitats with greater water availability, while species with a low seed E:Sarea frequently occupy drier habitats [51]. Optimizing the application of pelleting for species a with high E:Sarea may require bespoke pelleting methods, such as including materials that generate more hospitable environments for the emergence of these species. Consistent with past studies, we also observed that seed width, seed area, and seed coat thickness were predictive of species emergence speed [31,40,41]. Specifically, small-seeded, narrow-seeded, and thinly coated species generally exhibited faster emergence when grown from pellets relative to bare seeds. This result is significant for restoration practices, as the establishment of small-seeded species is generally harder in seed-based restoration due to factors such as seed displacement from wind, limited nutrient reserves to support regeneration, increased exposure to predation, and desiccation [52,53,54]. If pelleting can improve the establishment of small-seeded species in the field, this will help facilitate biodiverse outcomes in seed-based restoration. Finally, we observed that graminoids and herbaceous forbs generally exhibited high amenability to pellets, suggesting that this type of SET could be used to support the restoration of biodiverse, underutilized understory species to achieve high-diversity plantings [11]. 

Across the diverse suite of 64 species trialed in this study, the emergence and survival among individuals grown from pellets were generally high. Equally, a large proportion of high (57%) and medium (28%) amenability rankings were observed across our suite of tested species. Still, species-specific differences in survival and emergence were observed, where some species exhibited higher emergence and survival from pellets (e.g., *Chloris truncata*) relative to bare seeds, while others exhibited lower (e.g., *Arthropodium milleflorum*) or neutral (*Geranium solanderi*) effects. Although species with positive or neutral responses to seed pelleting may be highly amenable, it should be noted that pelleting may not be a ‘one size fits all’ prescription. That is, species exhibiting a negative response to pelleting in this study should not be deemed as ‘non-amenable’ to pelletized SETs. These species may simply require further research and development to customize the technology to suit the species’ germination, emergence, and early growth requirements. For example, all of the Acacia species tested in this study exhibited lower performance when grown from pellets compared to bare seeds (comprising 67% of all species ranked with “low amenability”). This trend may be driven by the materials included in the specific pellet recipe tested, as past research has found that Acacia seedlings often fail to emerge from clay-abundant substrates (one of the main constituents of the pellets used in this study) due to poor water infiltration, surface compaction, and root penetration resistance [2,55]. However, the higher water holding capacity of clay may be an important determinant of seedling survival if this species were to be tested in the field where water resources are limited [2]. Testing the application of pellets across this suite of species in the field is therefore required to assess whether emergence and survival trade-offs exist between bare vs. pelletized seeds and the resultant impact these may have on plant establishment in real-world scenarios. 

## 4. Materials and Methods

### 4.1. Seed Preparation

The 64 species trialed (Table S1) are native to the Sydney Basin Bioregion of eastern New South Wales, Australia. Species were selected to maximize taxonomic and functional diversity whilst ensuring a range of seed sizes and morphotypes were represented to ensure seed trait variation. Two seed provenance locations were selected across four of the trialed species (referred to as provenance 1 and 2 throughout) (Table S1). Seeds were supplied by Greening Australia (Richmond, NSW, Australia), the Australian Tree Seed Centre (Canberra, ACT, Australia) (CSIRO) and the Australian PlantBank (The Royal Botanic Gardens and Domain Trust) (Mt Annan, NSW, Australia). All seeds were stored at 15–20 °C and 30–50% relative humidity prior to planting in the experiment to reduce the risk of contamination and/or moisture reabsorption.

#### 4.1.1. Breaking Dormancy

Seeds of dormant species were pretreated prior to direct planting or pelleting to cue germination according to the methods outlined in [56,57] (Table S1). Seeds of Fabaceous species were submerged in boiling water (approximately 95 °C) for one minute then cooled under running water. All other physically dormant species were scarified by gently rubbing seeds between two sheets of fine-grit sandpaper. Physiologically dormant and/or smoke-responsive species were soaked in a 1:10-diluted solution of liquid smoke (Regen2000 Smokemaster, Grayson Australia Tecnica Pty Ltd., Brighton, Australia) for 12 h. Wet-treated seeds were dried at ambient temperature overnight. 

#### 4.1.2. Pellet Preparation

Two seed treatments were compared per species and provenance: (1) pellets and (2) bare seeds. Each experimental replicate consisted of one pellet and one bare seed replicate, each containing three seeds from the same species and seed lot, as informed by [23]. Pellet composition was informed by a commercial in confidence recipe developed by the Australian revegetation company AirSeed Technologies Australia Pty Ltd., Sydney, Australia (AirSeed). Pellets were made by combining dry ingredients (soil, compost, clay, charcoal) with water into a dough-like mixture, which were then hand-formed into smooth 20 mm-diameter spheres, the same size and shape as the pellets manufactured by AirSeed. Three seeds were sown on the pellet periphery at three evenly spaced points (approximately 0.5 cm from one another) to depths equaling the diameter of each seed, which were then covered by carefully smoothing the pellet surface. Pellets were dried at ambient temperature (22 °C) for 48 h prior to planting. Bare seeds were sown directly into the potting substrate at depths equaling the diameter of each seed at three evenly spaced points (approximately 0.5 cm from one another).

### 4.2. Greenhouse Experiment

The trial was conducted from March (autumn) to July (winter) 2023 within an irrigated greenhouse exposed to ambient temperature and relative humidity, located in Dural, NSW, Australia. For each species and seed provenance (n = 68), two polyethylene propagation trays (48 cm × 30 cm × 80 cm) were filled with a 1:1:1 ratio by volume mix of coconut fiber (GrowRite Pty Ltd., Sydney, NSW, Australia), potting mix (Langlands landscape supplies Pty Ltd., Dural, NSW, Australia), and medium-grain Chillagoe perlite (Ausperl Pty Ltd., Padstow, NSW, Australia). Bare and pelleted seed replicates (n = 50 per seed treatment, per species/provenance) were sown equidistantly (approximately 2 cm apart) in a 5 × 10 grid within each tray (Figure S1). Pellets were placed directly onto the substrate surface. Bare seeds, consisting of three ‘grouped’ seeds per replicate, were sown in three individual divots (approximately 2 cm apart) at the depth of the diameter of each seed, which were then covered by re-leveling the substrate surface. Trays were randomly positioned within the greenhouse and were re-randomized every seven days to minimize blocking effects. Trays were watered daily via an automated overhead mister system, adjusted for weather conditions and water requirements (i.e., lower water requirements in winter). From March to June, trays were watered three times a day for 3 min at each watering. From June to July (winter), trays were watered once a day for five minutes. 

### 4.3. Data Collection

Seedling emergence (defined as the first seedling per replicate to successfully penetrate the growth substrate or pellet surface) was scored weekly for each species and provenance from the date of planting and continued until emergence plateaued to zero for all species for 4 weeks (total data collection period was 18 weeks) as informed by [23]. When scoring emergence, only the first-emerged seedling of each grouped replicate of 3 seeds was counted. Survival was recorded across all emerged replicates for each species/provenance four weeks after each species’ emergence plateaued. The following emergence parameters were calculated for each seed treatment across all species/provenances at the close of the trial using methods from [58]: total emergence (%), time to emergence (minimum, mean, and maximum; days), emergence rate index (days), and time spread of emergence (days).

Seed trait measurements were obtained from images taken using a precision cabinet X-ray (KUBTEC Scientific, Stratford, CT, USA) at 4× magnification and processed using ImageJ software (version 1.54g) [59]. Fifty individual seeds were measured per species. The variables measured were seed length (mm), seed width (mm), seed coat thickness (mm), endosperm area (mm^2^), seed area (mm^2^), and circularity (ratio from 0 to 1; 1 corresponding to a perfect circle) using the methods described in [60]. Using these variables, seed shape variance was calculated using the length, width, and height of each seed, as described by [61]. Endosperm area to seed area ratio (E:Sarea) was calculated as a fraction of the estimated endosperm to seed area derived from our ImageJ processing methods described above. 

### 4.4. Statistical Analyses

Statistical analyses and figures were generated using R version 4.3.1 [62]. We used the ‘ggplot2’ [63] ‘car’ [64], ‘stats’ [62], ‘dplyr’ [65], and ‘lme4’ [66] packages to conduct analyses and visualize results. All linear mixed models (LMMs) were fit with the function ‘lmer’, and all ANOVA statistics were obtained with the function ‘anova’. All pairwise comparisons were calculated using the function ‘emmeans’ [67]. 

#### 4.4.1. Emergence Experiment

Pearson’s chi squared test of independence was used to assess differences in total seedling emergence (%) and survival (% replicates survived based on the total n that emerged) between treatments (bare seed vs. pellets), with each species analyzed separately. To evaluate differences in average time to emergence, we fit an LMM with species and treatment as fixed effects and replicate ID nested within species or provenance (where applicable) as a random effect. Species where no emergence or >3 replicates for a particular treatment was recorded were dropped from this analysis. LMMs were also fitted to evaluate differences in the emergence rate index and the time spread of emergence, where treatment was included as a fixed effect and species as a random effect (note: replicate ID could not be included as a fixed effect for these metrics given that they were calculated per seed lot, not replicate). 

#### 4.4.2. Characterizing Performance of Pellets

To explore how pelleting influenced species general amenability to this type of SET, pairwise comparisons from LMMs were used to summarize the effect of treatment (pellet vs. bare seed) on emergence and survival within species. For each response (emergence, survival), species with significantly improved or nonsignificantly different outcomes when grown from pellets relative to bare seeds were deemed as “highly-amenable” to pellets. Species with significantly improved or non-significantly different outcomes for one response variable and a significantly worse outcome for the other were ranked as “medium amenability”. Species with significantly worse outcomes across the two response variables were ranked with “low amenability” using the chosen pelleting method. Details of the categorization can be found in Table S2.

To increase the predictability of emergence outcomes when using pellets, species performance was also ranked to evaluate differences in emergence speed when grown from pellets relative to bare seeds. A ranking of “faster”, “slower”, or “equal” was assigned to each species for the three response variables (time to emergence, emergence rate index, and time spread of emergence) (Table S2). Pairwise comparisons were used to rank average time to emergence among species with significantly faster or slower emergence from pellets relative to bare seeds. Species with a higher emergence rate index or shorter time spread of emergence, when sown as pellets relative to bare seeds, were ranked as responding with faster emergence outcomes. Using these results, an overall emergence speed ranking was assigned for each species consisting of “faster” (faster emergence across at least two response variables), “slower” (slower emergence across at least two response variables), or “equal” (no difference compared to bare-seeded counterparts for at least two response variables) (Table S2).

Linear regressions were then used to explore whether morphological seed traits (seed length, seed width, seed area, seed coat thickness, E:Sarea, circularity) could predict species performance in pellets (using the ranked metrics for overall amenability and emergence speed). All univariate traits were independently evaluated against each performance ranking (overall amenability vs. emergency speed) with significant correlations described in Section 2.4 of the results. 

## 5. Conclusions

Successful restoration relies on the sustainable and effective use of native seeds when conducting revegetation [3,64]. The emerging applications of SETs, such as pelleting, using native species show promise in overcoming some barriers to restoration; yet, the knowledge of how diverse native species respond to seed enhancement is limited [22,23,68]. We demonstrate that some types of seed from native species are highly amenable to pelletization and that seed pelleting modifies emergence speed for many species. Morphological seed traits, particularly seed E:Sarea, seed width, seed area, and seed coat thickness, demonstrate patterns related to species performance in pellets and potentially provide the grounds to predict how different seed types perform as pelletized SETs in a greenhouse setting. Together, these results advance our understanding of the application of SETs across diverse native species and of how the integration of seed trait data can be used to target species for inclusion in ecological restoration using SETs.

## Figures and Tables

**Table 1:** Summary of performance results by species, treatment (bare seed vs. pellet), provenance (seed collected from two locations: 1, 2) (where applicable), and growth form (tree, shrub, herb, graminoid). Data presented include performance metrics associated with overall amenability to pelleting including the percent of replicates (n = 50) that emerged and survived prior to the conclusion of the experiment (18 weeks) as well as metrics associated with the speed of emergence, including the average time to emergence (days), emergence rate index (% per day), and the time spread of emergence (days) for each seed lot. Survival was calculated as the proportion of emerged replicates that were still alive following the species emergence plateau relative to the total number of recorded emergences. Grey bars indicate where no emergence was recorded. Species where no emergence was recorded across both treatments were removed.

Amenability Metric		Amenability					Emergence Speed					
			Total Emergence		Survival		Average Emergence		Emergence Rate Index		Time Spread of Emergence	
			(%)		(%)		(Days)		(%/Day)		(Days)	
Species	Provenance	Growth Form	Bare Seed	Pellet	Bare Seed	Pellet	Bare Seed	Pellet	Bare Seed	Pellet	Bare Seed	Pellet
*Acacia decurrens*		Shrub	100	70	100	66	14	25	3.57	0.49	0	16
*Acacia falcata*	1	Shrub	94	48	100	80	23	35	0.73	0.14	16	27
*Acacia falcata*	2	Shrub	90	30	98	96	20	32	0.6	0.11	24	42
*Acacia implexa*		Shrub	98	66	100	76	15	27	1.11	0.26	16	42
*Acacia parramattensis*		Shrub	94	60	100	67	15	22	1.59	0.46	8	24
*Ajuga australis*		Herb	66	22	97	100	36	34	0.16	0.06	94	36
*Allocasuarina littoralis*		Tree	88	66	100	100	21	34	0.46	0.21	43	49
*Aristida vagans*		Graminoid	30	22	73	64	21	27	0.2	0.13	27	78
*Arthropodium milleflorum*		Herb	88	48	100	79	32	48	0.22	0.08	42	120
*Bothriochloa macera*		Graminoid	54	58	93	90	17	21	0.53	0.39	13	64
*Bursaria spinosa*		Shrub	58	100	90	100	36	28	0.15	0.6	89	12
*Carex inversa*		Graminoid	50	98	96	82	48	35	0.08	0.21	65	41
*Centella asiatica*		Herb	30	82	80	73	38	34	0.08	0.27	78	34
*Chenopodium nutans*		Herb	6	42	67	76	17	34	0.09	0.12	8	113
*Chenopodium trigonon*		Herb	80	100	35	94	23	14	0.39	1.86	113	7
*Chloris truncata*		Graminoid	40	62	35	77	24	13	0.19	0.97	50	15
*Chloris ventricosa*		Graminoid	24	20	100	60	13	17	0.39	0.16	15	31
*Coleus australis*		Shrub	10	82	100	95	26	24	0.1	0.32	12	41
*Commelina cyanea*		Herb	24	52	92	96	20	24	0.21	0.28	20	27
*Convolvulus erubescens*		Shrub	40	16	90	100	8	15	1.37	0.51	23	64
*Corymbia maculata*		Tree		4		100		69		0.02		51
*Cymbopogon refractus*		Graminoid	46	34	74	76	17	27	0.46	0.17	13	20
*Daviesia ulicifolia*		Shrub	58	62	100	45	31	37	0.25	0.14	29	105
*Dillwynia sieberi*		Shrub	96	72	100	78	27	49	0.33	0.12	43	105
*Dodonaea viscosa*	1	Shrub	62	4	100	100	23	111	0.01	0.12	33	94
*Dodonaea viscosa*	2	Shrub	28	4	84	100	35	30	0.12	0.06	94	0
*Eremophila debilis*		Herb	16	32	88	81	40	64	0.07	0.06	12	56
*Eucalyptus amplifolia*		Tree	84	100	95	100	20	10	0.51	2.81	105	8
*Eucalyptus crebra*	1	Tree	78	86	97	88	10	19	1.28	0.66	36	80
*Eucalyptus crebra*	2	Tree	100	100	98	100	16	15	0.83	0.89	36	23
*Eucalyptus eugenioides*		Tree	66	88	100	95	18	19	0.94	1.23	8	8
*Eucalyptus longifolia*		Tree	42	92	90	100	27	20	0.21	0.83	34	41
*Eucalyptus moluccana*	1	Tree	86	82	100	95	14	14	0.92	0.84	57	36
*Eucalyptus moluccana*	2	Tree	100	100	100	92	13	12	1.31	1.58	50	31
*Eucalyptus punctata*		Tree	86	44	93	100	25	34	0.58	0.25	12	33
*Eucalyptus tereticornis*		Tree	100	82	100	95	10	12	2.07	1.41	36	15
*Fimbristylis dichotoma*		Graminoid	32	98	44	59	36	26	0.12	0.97	26	12
*Geranium solanderi*		Herb	94	88	100	89	20	18	0.56	0.65	31	24
*Glossocardia bidens*		Herb	34	40	53	50	23	32	0.25	0.12	13	72
*Hardenbergia violacea*		Shrub	84	74	100	81	21	25	0.38	0.32	43	29
*Hypericum gramineum*		Herb	96	52	81	58	31	23	0.34	0.58	57	16
*Indigofera australis*		Shrub	82	20	71	70	12	16	1.05	0.19	23	23
*Laxmannia gracilis*		Herb	60	6	100	100	44	45	0.11	0.03	78	7
*Lomandra longifolia*		Graminoid	30		73		74		0.07		0.37	
*Melaleuca decora*		Shrub	38	100	84	86	39	28	0.14	0.98	56	56
*Mentha satureioides*		Herb	2	4	100	0	57	44	0.02	0.07	0	72
*Microlaena stipoides*		Graminoid	20		60		17		0.3		8	
*Ozothamnus diosmifolius*		Shrub	32	12	69	0	13	8	0.51	0.75	21	0
*Panicum simile*		Graminoid	4	20	50	40	22	31	0.09	0.09	0	49
*Pentapogon micranthus*		Graminoid	92	100	87	100	21	17	0.5	1	41	20
*Phyllanthus virgatus*		Herb		8		75		47		0.04		7
*Plantago gaudichaudii*		Herb	52	94	96	100	21	13	0.3	1.34	72	16
*Poa labillardierei*		Graminoid	78	56	90	46	17	14	0.62	2	24	0
*Pomax umbellata*		Shrub	12	52	0	69	73	63	0.03	0.1	37	28
*Sarga leioclada*		Graminoid	8	4	75	100	19	33	0.11	0.04	13	34
*Senecio quadridentatus*		Herb	8		75		33		0.04		34	
*Setaria distans*		Graminoid	38	24	58	83	28	43	0.18	0.06	26	97
*Solanum prinophyllum*		Herb	74	80	95	88	19	26	0.7	0.33	16	29
*Sporobolus creber*		Graminoid	24	98	83	98	31	20	0.09	1.28	41	8
*Syncarpia glomulifera*		Tree	30	6	80	100	31	36	0.14	0.04	35	21
*Themeda triandra*		Graminoid	16	12	100	100	10	14	0.46	0.43	7	0
*Wahlenbergia gracilis*		Herb	86	54	72	37	33	22	0.25	0.61	57	8

## Data Availability

The original data presented in the study are openly available at https://osf.io/5wc4q/ at DOI 10.17605/OSF.IO/5WC4Q (accessed on 6 June 2024).

## Supplementary Materials

The following supporting information can be downloaded at: https://www.mdpi.com/article/10.3390/plants13162256/s1, Figure S1: Planting design of (A) pelleted seeds and (B) bare seeds within the two propagation trays allocated to each species/provenance (n = 50 replicates per seed treatment, per species/provenance). Table S1: List of 64 native Australian plant species (and their provenance and/or seed pre-treatment, where applicable) used in the experimental trial of seed pelleting technology. Table S2: Outcomes of encapsulation in pellets for the 58 species tested in this study. Species with no emergence (n = 6) were removed from this table. Responses were ranked to yield two general performance metrics: (1) an overall amenability to pellets; “high” (amenable across two response variables), “medium” (amenable across one response variable), and “low” (not amenable based on either response variable), and (2) an estimate of emergence speed; “faster” (faster than directly down counterparts for at least two emergence speed metrics), “slower” (slower than directly down counterparts for at least two emergence speed metrics), “no difference” (no difference compared to directly down counterparts for at least two emergence speed metrics). Table S3: Results of Pearson’s chi squared (X^2^) tests of independence comparing seedling emergence counts. Contrasts compared between treatments (bare seeds-seed pellets, n = 50) for each species included in the experiment *** significant at 0.001, ** significant at 0.01, * significant at 0.05. Grey bars indicate where chi-squared could not be computed as no emergence or survival was recorded for a particular species or treatment. Table S4: Estimated marginal means contrasting experimental seed treatment effects (pellet-bare seed) on average time to emergence (days) within species using the linear mixed effects model. Contrasts compared between treatments (bare seeds-seed pellets, n = 50) for each species included in the experiment *** significant at 0.001, ** significant at 0.01, * significant at 0.05.
