# Supplementary material for: Morphological Seed Traits Predict Early Performance of Native Species to Pelletized Seed Enhancement Technologies
Source: Plants (Basel). 2024 Aug 14;13(16):2256. doi: 10.3390/plants13162256 (PMC11360080; doi:10.3390/plants13162256)
Supplement: Supplementary file 1 [file plants-13-02256-s001.zip › plants-3078474-supplementary.pdf]

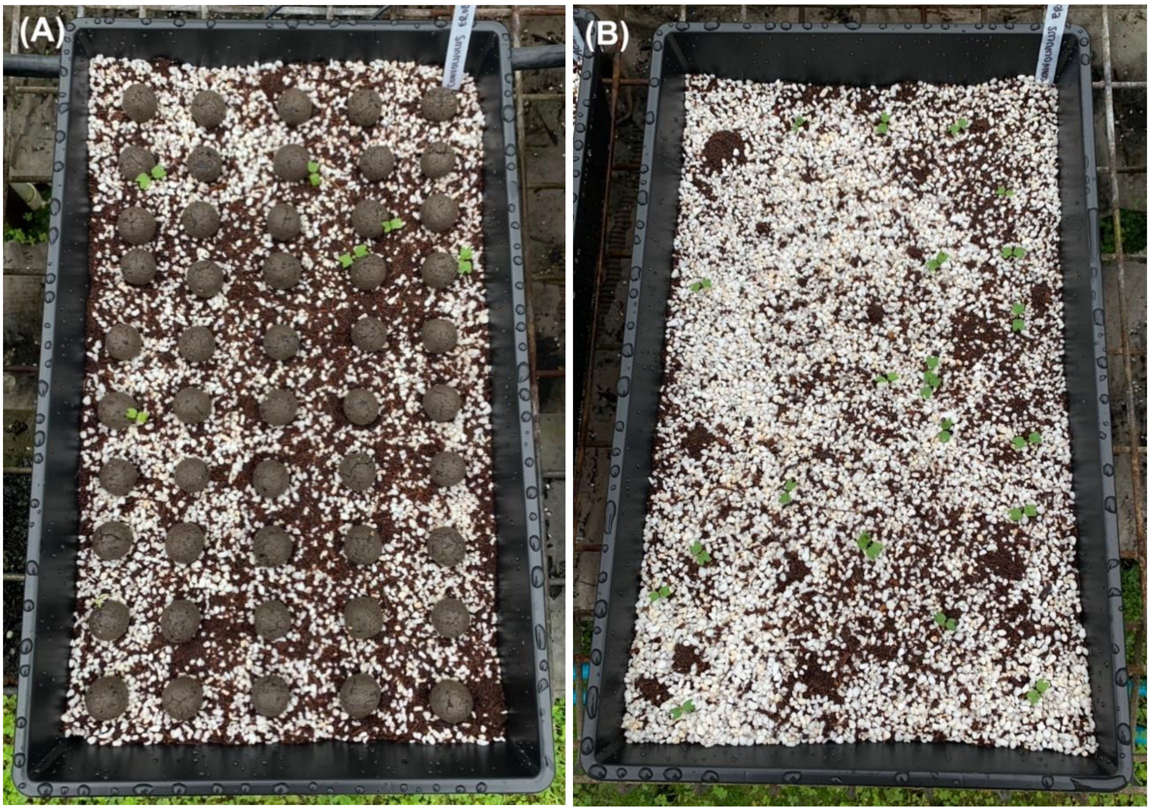

Figure S1. Planting design of (A) pelleted seeds and (B) bare seeds within the two propagation trays allocated to each species/provenance (n = 50 replicates per seed treatment, per species/provenance).

SUPPLEMENTARY INFORMATION

Table S1 List of 64 native Australian plant species (and their provenance and/or seed pre-treatment, where applicable) used in the experimental trial of seed pelleting technology.

**Species**	**Family**	**Provenance**	**Seed pre-treatment**
Acacia decurrens	Fabaceae		Wet heat
Acacia falcata	Fabaceae	2 + 1	Wet heat
Acacia implexa	Fabaceae		Wet heat
Acacia parramattensis	Fabaceae		Wet heat
Ajuga australis	Lamiaceae		Smoke
Allocasuarina littoralis	Casuarinaceae		Smoke
Aristida vagans	Poaceae		
Arthropodium milleflorum	Asparagaceae		Smoke
Bothriochloa macra	Poaceae		
Bursaria spinosa	Pittosporaceae		
Caesia parviflora	Asphodelaceae		
Carex inversa	Cyperaceae		
Centella asiatica	Apiaceae		
Chloris truncata	Poaceae		Smoke
Chloris ventricosa	Poaceae		Smoke
Commelina cyanea	Commelinaceae		
Convolvulus erubescens	Convolvulaceae		Scarify
Corymbia maculata	Myrtaceae		
Cymbopogon refractus	Poaceae		
Daviesia ulicifolia	Fabaceae		Wet heat
Dichelachne micrantha	Poaceae		
Dillwynia sieberi	Fabaceae		Wet heat
Dodonaea viscosa	Sapindaceae	2 + 1	Scarify
Einadia nutans	Amaranthaceae		
Einadia trigonos	Amaranthaceae		
Eragrostis brownii	Poaceae		
Eremophila debilis	Scrophulariaceae		Scarify
Eucalyptus amplifolia	Myrtaceae		
Eucalyptus crebra	Myrtaceae	2 + 1	
Eucalyptus eugenioides	Myrtaceae		
Eucalyptus globoidea	Myrtaceae		
Eucalyptus longifolia	Myrtaceae		

Eucalyptus moluccana	Myrtaceae	2 + 1	
Eucalyptus punctata	Myrtaceae		
Eucalyptus tereticornis	Myrtaceae		
Fimbristylis dichotoma	Cyperaceae		
Geranium solanderi	Geraniaceae		Scarify
Glossocardia bidens	Asteraceae		
Hardenbergia violacea	Fabaceae		Wet heat
Hypericum gramineum	Hypericaceae		Smoke
Indigofera australis	Fabaceae		Wet heat
Laxmannia gracilis	Asparagaceae		Smoke
Leucopogon juniperinus	Ericaceae		Smoke
Lomandra longifolia	Asparagaceae		
Melaleuca decora	Myrtaceae		
Mentha satureioides	Lamiaceae		
Microlaena stipoides	Poaceae		
Ozothamnus diosmifolius	Asteraceae		
Panicum simile	Poaceae		Smoke
Paspalidium distans	Poaceae		
Phyllanthus virgatus	Phyllanthaceae		
Plantago gaudichaudii	Plantaginaceae		
Plectranthus parviflorus	Lamiaceae		
Poa labillardierei	Poaceae		Smoke
Pomax umbellata	Rubiaceae		
Senecio quadridentatus	Asteraceae		
Solanum prinophyllum	Solanaceae		Smoke
Solenogyne bellioides	Asteraceae		
Sorghum leiocladum	Poaceae		
Sporobolus creber	Poaceae		
Syncarpia glomulifera	Myrtaceae		
Themeda triandra	Poaceae		Smoke
Vittadinia cuneata	Asteraceae		
Wahlenbergia gracilis	Campanulaceae		Smoke

Table S2 Outcomes of encapsulation in pellets for the 58 species tested in this study. Species with no emergence (n = 6) were removed from this table. Responses were ranked to yield two general performance metrics: (1) an overall amenability to pellets; "high" (amenable across two response variables), "medium" (amenable across one response variable), and "low" (not amenable based on either response variable), and (2) an estimate of emergence speed; "faster" (faster than directly down counterparts for at least two emergence speed metrics), "slower" (slower than directly down counterparts for at least two emergence speed metrics), "no difference" (no difference compared to directly down counterparts for at least two emergence speed metrics).

**Species**	**Amenability**	**Emergence speed**
Bursaria spinosa	High	Faster
Carex inversa	High	Faster
Centella asiatica	High	Faster
Chloris truncata	High	Faster
Dichelachne micrantha	High	Faster
Eucalyptus amplifolia	High	Faster
Eucalyptus longifolia	High	Faster
Fimbristylis dichotoma	High	Faster
Geranium solanderi	High	Faster
Melaleuca decora	High	Faster
Plantago guadichaudii	High	Faster
Pomax umbellata	High	Faster
Sporobolus creber	High	Faster
Commelina cyanea	High	No difference
Corymbia maculata	High	No difference
Eucalyptus eugenioides	High	No difference
Mentha satureioides	High	No difference
Panicum simile	High	No difference
Themeda triandra	High	No difference
Aristida vagans	High	Slower
Bothriochloa macra	High	Slower
Chloris ventricosa	High	Slower
Cymbopogon refractus	High	Slower

Einadia nutans	High	Slower
Eremophila debilis	High	Slower
Eucalyptus crebra	High	Slower
Glossocardia bidens	High	Slower
Paspalidium distans	High	Slower
Solanum prinophyllum	High	Slower
Sorghum leiocladum	High	Slower
Einadia trigonos	Med	Faster
Eucalyptus moluccana	Med	Faster
Hypericum gramineum	Med	Faster
Ajuga australis	Med	No difference
Eucalyptus tereticornis	Med	No difference
Indigofera australis	Med	No difference
Laxmannia gracilis	Med	No difference
Plectranthus parviflorus	Med	No difference
Syncarpia glomulifera	Med	No difference
Allocasuarina littoralis	Med	Slower
Convolvulus erubescens	Med	Slower
Daviesia ulicifolia	Med	Slower
Dodonaea viscosa	Med	Slower
Eucalyptus punctata	Med	Slower
Hardenbergia violacea	Med	Slower
Poa labillardierei	Low	Faster
Wahlenbergia gracilis	Low	Faster
Acacia decurrens	Low	Slower
Acacia falcata	Low	Slower
Acacia implexa	Low	Slower
Acacia parramattensis	Low	Slower
Arthropodium milleflorum	Low	Slower
Dillwynia sieberi	Low	Slower

Table S3 Results of Pearson's chi squared (X^2) tests of independence comparing seedling emergence counts. Contrasts compared between treatments (bare seeds-seed pellets, n = 50) for each species included in the experiment *** significant at .001, ** significant at .01, * significant at .05. Grey bars indicate where chi-squared could not be computed as no emergence or survival was recorded for a particular species or treatment.

		**Emerged**		**Survived**	
**Species**	**df**	X^2	***P***	X^2	***P***
Acacia decurrens	1	15.37	<0.001***	17.23	<0.001***
Acacia falcata	1	59.83	<0.001***	4.02	0.045*
Acacia implexa	1	15.24	<0.001***	10.55	0.002**
Acacia parramattensis	1	14.46	<0.001***	15.18	<0.001***
Ajuga australis	1	17.90	<0.001***	0.00	1.000
Allocasuarina littoralis	1	5.65	0.018*	0.00	1.000
Aristida vagans	1	0.47	0.494	0.01	0.921
Arthropodium milleflorum	1	16.59	<0.001***	7.07	0.008**
Bothriochloa macra	1	0.04	0.840	0.00	1.000
Bursaria spinosa	1	24.11	<0.001***	2.92	0.088
Caesia parviflora	1				
Carex inversa	1	27.50	<0.001***	1.82	0.177
Centella asiatica	1	25.37	<0.001***	0.03	0.862
Chloris truncata	1	4.00	0.045*	7.48	0.006**
Chloris ventricosa	1	0.06	0.809	3.49	0.062
Commelina cyanea	1	7.17	0.007**	0.00	1.000
Convolvulus erubescens	1	6.00	0.014*	0.01	0.908
Corymbia maculata	1	0.51	0.475	0.00	1.000
Cymbopogon refractus	1	1.04	0.307	0.00	1.000
Daviesia ulicifolia	1	0.04	0.838	19.57	<0.001***
Dichelachne micrantha	1	2.34	0.126	0.49	0.027*
Dillwynia sieberi	1	9.00	0.003**	9.35	0.002**
Dodonaea viscosa	1	43.25	<0.001***	0.00	1.000
Einadia nutans	1	15.84	<0.001***	0.00	1.000
Einadia trigonos	1	9.00	0.003**	32.77	<0.001***
Eragrostis brownii	1				
Eremophila debilis	1	2.69	0.101	0.00	1.000
Eucalyptus amplifolia	1	6.66	0.010*	0.71	0.400
Eucalyptus crebra	1	0.55	0.459	0.51	0.477
Eucalyptus eugenioides	1	5.65	0.017*	0.27	0.605

Eucalyptus globoidea	1				
Eucalyptus longifolia	1	26.05	<0.001***	1.83	0.177
Eucalyptus moluccana	1	0.07	0.794	4.42	0.036*
Eucalyptus punctata	1	17.58	<0.001***	0.41	0.520
Eucalyptus tereticornis	1	7.81	0.005*	0.74	0.389
Fimbristylis dichotoma	1	45.01	<0.001***	0.62	0.430
Geranium solanderi	1	0.49	0.485	3.67	0.055
Glossocardia bidens	1	0.17	0.679	0.00	1.000
Hardenbergia violacea	1	0.96	0.326	6.53	0.011*
Hypericum gramineum	1	22.92	<0.001***	3.63	0.057
Indigofera australis	1	36.01	<0.001***	0.00	1.000
Laxmannia gracilis	1	30.57	<0.001***	0.00	1.000
Leucopogon juniperinus	1				
Lomandra longifolia	1	15.37	<0.001***		
Melaleuca decora	1	42.08	<0.001***	0.00	1.000
Mentha satureioides	1	0.00	1.000	0.19	0.665
Microlaena stipoides	1	9.00	0.003**		
Ozothamnus diosmifolius	1	4.72	0.030*	5.73	0.017*
Panicum simile	1	4.64	0.031*	0.00	1.000
Paspalidium distans	1	1.68	0.195	1.17	0.280
Phyllanthus virgatus	1	2.34	0.126		
Plantago guadichaudii	1	20.29	<0.001***	0.09	0.726
Plectranthus parviflorus	1	49.32	<0.001***	0.00	1.000
Poa labillardierei	1	4.52	0.033*	13.00	<0.001***
Pomax umbellata	1	16.59	<0.001***	6.89	0.009**
Senecio quadridentatus	1	2.34	0.126		
Solanum prinophyllum	1	0.23	0.635	0.47	0.493
Solenogyne bellioides	1				
Sorghum leiocladum	1	0.18	0.674	0.00	1.000
Sporobolus creber	1	54.48	<0.001***	1.84	0.175
Syncarpia glomulifera	1	8.20	0.004**	0.00	1.000
Themeda triandra	1	0.08	0.773		
Vittadinia cuneata	1				
Wahlenbergia gracilis	1	10.71	<0.001***	7.02	0.008**

Table S4 Estimated marginal means contrasting experimental seed treatment effects (pellet-bare seed) on average time to emergence (days) within species using the linear mixed effects model. Contrasts compared between treatments (bare seeds-seed pellets, n = 50) for each species included in the experiment *** significant at .001, ** significant at .01, * significant at .05

Species	Contrast	Estimate	SE	*p*
Acacia decurrens	Bare seed - Pellet	-10.514	2.588	<0.001***
Acacia falcata	Bare seed - Pellet	-12.240	2.244	<0.001***
Acacia implexa	Bare seed - Pellet	-11.985	2.644	<0.001***
Acacia parramattensis	Bare seed - Pellet	-7.075	2.744	0.010*
Ajuga australis	Bare seed - Pellet	1.909	4.088	0.641
Allocasuarina littoralis	Bare seed - Pellet	-12.788	2.704	<0.001***
Aristida vagans	Bare seed - Pellet	-6.448	4.661	0.167
Arthropodium milleflorum	Bare seed - Pellet	-16.284	2.980	<0.001***
Bothriochloa macra	Bare seed - Pellet	-4.009	3.140	0.202
Bursaria spinosa	Bare seed - Pellet	8.225	2.741	0.003**
Carex inversa	Bare seed - Pellet	12.592	2.886	<0.001***
Centella asiatica	Bare seed - Pellet	4.320	3.544	0.223
Chloris truncata	Bare seed - Pellet	11.261	3.368	<0.001***
Chloris ventricosa	Bare seed - Pellet	-3.533	5.028	0.482
Commelina cyanea	Bare seed - Pellet	-4.410	4.098	0.282
Convolvulus erubescens	Bare seed - Pellet	-6.850	4.912	0.163
Cymbopogon refractus	Bare seed - Pellet	-9.445	3.756	0.012*
Daviesia ulicifolia	Bare seed - Pellet	-6.502	3.034	0.032*
Dichelachne micrantha	Bare seed - Pellet	3.590	2.399	0.135
Dillwynia sieberi	Bare seed - Pellet	-21.972	2.589	<0.001***
Dodonaea viscosa	Bare seed - Pellet	-43.161	6.127	<0.001***
Einadia nutans	Bare seed - Pellet	-17.190	7.248	0.018*
Einadia trigonos	Bare seed - Pellet	9.330	2.491	<0.001***
Eremophila debilis	Bare seed - Pellet	-23.375	5.085	<0.001***
Eucalyptus amplifolia	Bare seed - Pellet	10.210	2.458	<0.001***
Eucalyptus crebra	Bare seed - Pellet	-3.071	1.741	0.078
Eucalyptus eugenioides	Bare seed - Pellet	-0.364	2.704	0.893
Eucalyptus longifolia	Bare seed - Pellet	6.962	3.093	0.024*
Eucalyptus moluccana	Bare seed - Pellet	0.574	1.732	0.740
Eucalyptus punctata	Bare seed - Pellet	-9.073	3.078	0.003**
Eucalyptus tereticornis	Bare seed - Pellet	-1.281	2.474	0.605
Fimbristylis dichotoma	Bare seed - Pellet	9.791	3.381	0.004**
Geranium solanderi	Bare seed - Pellet	2.126	2.463	0.388
Glossocardia bidens	Bare seed - Pellet	-8.306	3.874	0.032*
Hardenbergia violacea	Bare seed - Pellet	-4.024	2.648	0.129
Hypericum gramineum	Bare seed - Pellet	8.385	2.859	0.003**
Indigofera australis	Bare seed - Pellet	-4.068	4.142	0.326
Laxmannia gracilis	Bare seed - Pellet	-1.267	7.111	0.859
Melaleuca decora	Bare seed - Pellet	10.888	3.165	<0.001***
Mentha satureioides	Bare seed - Pellet	13.000	14.382	0.366

Ozothamnus diosmifolius	Bare seed - Pellet	5.437	5.622	0.333
Panicum simile	Bare seed - Pellet	-8.600	9.096	0.344
Paspalidium distans	Bare seed - Pellet	-15.338	4.330	<0.001***
Plantago guadichaudii	Bare seed - Pellet	7.322	2.870	0.011*
Plectranthus parviflorus	Bare seed - Pellet	2.741	5.563	0.622
Poa labillardierei	Bare seed - Pellet	3.487	2.909	0.231
Pomax umbellata	Bare seed - Pellet	9.577	5.319	0.072
Solanum prinophyllum	Bare seed - Pellet	-6.802	2.679	0.011
Sorghum leiocladum	Bare seed - Pellet	-13.750	10.170	0.176
Sporobolus creber	Bare seed - Pellet	10.915	3.782	0.004
Syncarpia glomulifera	Bare seed - Pellet	-4.667	7.427	0.530
Themeda triandra	Bare seed - Pellet	-4.375	6.342	0.490
Wahlenbergia gracilis	Bare seed - Pellet	10.704	2.883	0.000
